# What is the most cost‐effective strategy for nasal screening and *Staphylococcus aureus* decolonization in patients undergoing total hip arthroplasty?

**DOI:** 10.1186/s12891-021-04008-y

**Published:** 2021-02-01

**Authors:** Hisahiro Tonotsuka, Hajime Sugiyama, Ayano Amagami, Keigo Yonemoto, Ryuichi Sato, Mitsuru Saito

**Affiliations:** 1 Department of Orthopaedic Surgery, Kanagawa Rehabilitation Hospital, 516 Nanasawa, Kanagawa 516, 243-0121 Atsugi, Japan; 2 Department of Orthopaedic Surgery, The Jikei University School of Medicine, 3-19-18 Nishishinbashi, Minato-ku 105-8471 Tokyo, Japan

**Keywords:** Total hip arthroplasty, *Staphylococcus aureus*, Periprosthetic joint infection, Nasal decolonization, Screening

## Abstract

**Background:**

To reduce periprosthetic joint infection after total hip arthroplasty (THA), several nasal screening and decolonization strategies for methicillin-resistant *Staphylococcus aureus* (MRSA) and methicillin-sensitive *Staphylococcus aureus* (MSSA) have been performed. These include universal decolonization (UD; i.e., no screening and decolonization for all patients), universal screening and target decolonization (US; i.e., screening for all patients and decolonization for bacterial positive patients), and target screening and decolonization (TS; i.e., screening and decolonization for high-risk populations only). Although TS is the most cost-effective strategy, useful risk factors must be identified. The purpose of this study was to evaluate the presence of predictive factors that enable the TS strategy to be successfully implemented and to compare the costs of each strategy.

**Methods:**

A total of 1654 patients scheduled for primary or revision THA (1464 female, 190 male; mean age 64 years) were screened prior to surgery for bacterial colonization of the nasal mucosa. Risk factors for positive MRSA and *S. aureus* (including both MRSA and MSSA) tests were analyzed according to the following parameters: sex, age ≥ 80 years, body mass index ≥ 30 kg/m^2^, antibiotic use within 3 years, corticosteroid use, serum albumin < 3.5 g/dL, glomerular filtration rate < 50 mL/min, presence of brain, thyroid, cardiac, or pulmonary disease, diabetes, asthma, smoking status, and whether revision surgery was performed. The average cost of each strategy was calculated.

**Results:**

In total, 29 patients (1.8 %) tested positive for MRSA and 445 (26.9 %) tested positive for *S. aureus*. No parameters were identified as independent risk factors for MRSA and only female sex was identified as a risk factor for *S. aureus* (p = 0.003; odds ratio: 1.790; 95 % confidence interval: 1.210–2.640). The average cost of each strategy was 1928.3 yen for UD, 717.6 yen for US, and 717.6 yen for TS (for eradicating MRSA), and 1928.3 yen for UD, 1201.6 yen for US, and 1160.4 yen for TS (for eradicating *S. aureus*).

**Conclusions:**

No useful predictive parameters for implementing the TS strategy were identified. Based on cost implications, US is the most cost-effective strategy for THA patients.

## Background

Total hip arthroplasty (THA) is a common surgical procedure, and the number of such procedures performed is expected to increase with increasing aging of society [1]. Although THA is an effective procedure with a low complication rate, periprosthetic joint infection (PJI) following THA is a devastating complication. PJI from methicillin-resistant *Staphylococcus aureus* (MRSA) and methicillin-sensitive *Staphylococcus aureus* (MSSA) leads to increased mortality, longer hospitalization, and higher costs [2, 3]. Therefore, prevention and control strategies for PJI caused by MRSA and MSSA are critical for both patient safety and cost reduction.

One of the most important factors in reducing infections is identifying and eradicating nasal bacterial colonization prior to surgery. Previous studies have shown that preoperative nasal decolonization for *S. aureus* (including both MRSA and MSSA) in THA patients can reduce the risk of surgical site infection (SSI), which can lead to PJI [4, 5]. However, various screening and decolonization strategies have been reported: universal decolonization (UD), which involves no screening and decolonization of all patients [6, 7]; universal screening and target decolonization (US), which involves screening for all patients and decolonization for MRSA or *S. aureus*-positive patients [4, 5, 8, 9]; and target screening and decolonization (TS), which involves screening for high-risk populations only and decolonization for MRSA or *S. aureus*-positive patients [10]. Controversy remains as to the most appropriate strategy.

Although UD can significantly reduce the prevalence of *S. aureus* carriers without a waiting period for screening results [7], the protocol has problems of higher cost and the development of resistant strains [11]. US can reduce sampling error rates and offers a cost-effective screening method [4, 5]. One option to reduce the cost of screening is to implement TS based on certain ‘risk factors’. However, this strategy requires determining these risk factors with appropriate sensitivity and specificity. According to Dave et al., the TS method failed to detect half of the MRSA-positive cases in their study [8]. Thus, clarifying whether proper predetermined factors are correlated with the risk of *S. aureus* carrier status is important in TS. Moreover, although financial considerations are crucial for implementing nasal screening and decolonization, few reports have compared the relative costs of these three strategies after performing simulations and whether TS is actually able to reduce costs after searching for useful risk factors that allow this strategy to be implemented.

The purpose of this study was to investigate whether it is possible to determine the predictive factors for successful implementation of the TS strategy with *S. aureus* carriers, and to clarify the most cost-effective strategy for patients who undergo THA surgery.

## Methods

We evaluated 1654 patients (1464 female, 190 male; mean age at time of surgery 64 (21–89) years) who underwent primary or revision THA from March 2008 to December 2017 at our institution. The study included 1499 cases of primary THA and 155 cases of revision THA.

All patients were screened for nasal bacterial colonization with a sterile swab 1–4 weeks prior to surgery. Patients who tested positive for MRSA or MSSA were registered. Patients positive for MRSA were treated with mupirocin for both nares twice for 5 days. The prevalence of S. aureus and MRSA carriers was investigated, and all patients were divided into two groups based on the presence of MRSA—MRSA-positive (MR+) group and MRSA-negative (MR−) group—and then based on the presence of *S. aureus*—*S. aureus*-positive (SA+) group and *S. aureus*-negative (SA−) group. Risk factors for positive*S. aureus* and MRSA tests were analyzed according to the following parameters: sex, age at time of surgery (≥ 80 or < 80 years), body mass index (BMI: ≥30 or < 30 kg/m^2^), antibiotic use within 3 years before surgery, previous or present corticosteroid use, serum albumin (< 3.5 or ≥ 3.5 g/dL), glomerular filtration rate (GFR: <50 or ≥ 50 mL/min), presence or not of brain, thyroid, cardiac, or pulmonary disease, diabetes, asthma, smoking habit, and whether revision surgery was performed. Screening and decolonization costs for each strategy were calculated. Based on average figures for our institution, a surgeon’s hourly rate is 4666 yen, a medical assistant’s hourly rate is 1722 yen, and a laboratory technician’s hourly rate is 1758 yen. The time expenditure was as follows: selection of patients for screening using the TS strategy by the surgeon, 1 minute; assessment of the culture results by the surgeon, 1 minute; nasal swab sample obtained by a medical assistant, 1 minute; instructions on how to apply mupirocin ointment by a medical assistant, 10 minutes; and processing of each laboratory test by a laboratory technician, 5 minutes.

Fisher’s exact test was used for univariate analysis to identify risk factors for S. aureus and MRSA, and stepwise logistic regression was used for multivariate analysis.

## Results

### Number of cases for each parameter

The study included 1464 female patients (88.5 %) and 190 male patients (11.5 %). The number of cases for each parameter was as follows: age ≥ 80 years, 70 cases (4.2 %); BMI ≥ 30 kg/m^2^, 93 cases (5.6 %); previous antibiotic use, 337 cases (20.4 %); corticosteroid use, 36 cases (2.2 %); serum albumin < 3.5 g/dL, 18 cases (1.1 %); GFR < 50 mL/min, 108 cases (6.5 %); brain disease, 54 cases (3.3 %); thyroid disease, 29 cases (1.8 %); cardiac disease, 21 cases (1.3 %); pulmonary disease, 20 cases (1.2 %); diabetes, 109 cases (6.6 %); asthma, 55 cases (3.3 %); smoking habit, 76 cases (4.6 %); and revision surgery, 155 cases (9.4 %).

### Risk factors for MRSA

In total, 29 cases (1.8 %) were classified in the MR + group and 1625 in the MR − group. Univariate analysis found no significant difference between the proportion of female patients in the MR + group relative to all female patients (25 vs. 1464) and relative to all male patients (4 vs. 190) (1.7 % vs. 2.1 %, *p* = 0.567; odds ratio [OR]: 0.808; 95 % confidence interval [CI]: 0.275–3.229).

Similarly, as shown in Table 1, no significant differences were found between age ≥ 80 and < 80 years, BMI ≥ 30 and < 30 kg/m^2^, antibiotic use within 3 years or not, corticosteroid use or not, serum albumin < 3.5 and ≥ 3.5 g/dL, GFR < 50 and ≥ 50 mL/min, presence or not of brain, thyroid, cardiac, or pulmonary disease, diabetes or asthma, smoking habit, or whether revision surgery was performed.

Multivariate analysis revealed no parameters as independent risk factors.

**Table 1:** Univariate analysis of MR+ and MR- groups

							95% Confidence interval	
Parameter		n	MR+ (*n*=29)	MR- (*n*=1625)	*P*-value	Odds ratio	Lower	Upper
Sex	Female	1464	25 (1.7%)	1439 (98.3%)	0.567	0.808	0.275	3.229
	Male	190	4 (2.1%)	186 (97.9%)				
Age (years)	≥80	70	2 (2.9%)	68 (97.1%)	0.350	1.695	0.192	6.987
	<80	1584	27 (1.7%)	1557 (98.3%)				
BMI (kg/m^2^)	≥30	93	3 (3.2%)	90 (96.8%)	0.221	1.967	0.374	6.605
	<30	1561	26 (1.7%)	1535 (98.3%)				
Antibiotic use within 3 years	+	337	8 (2.4%)	329 (97.6%)	0.351	1.500	0.569	3.565
	-	1317	21 (1.6%)	1296 (98.4%)				
Corticosteroid use	+	36	2 (5.6%)	34 (94.4%)	0.130	3.461	0.384	14.752
	-	1618	27 (1.7%)	1591 (98.3%)				
Albumin (g/dL)	<3.5	18	1 (5.6 %)	17 (94.4 %)	0.274	3.374	0.078	23.091
	≥3.5	1636	28 (1.7 %)	1608 (98.3 %)				
GFR (mL/min)	<50	108	3 (2.8%)	105 (97.2%)	0.432	1.670	0.318	5.586
	≥50	1546	26 (1.7%)	1520 (98.3%)				
Brain disease	+	54	0 (0.0%)	54 (100.0%)	1.000	0.000	0.000	4.078
	-	1600	29 (1.8%)	1571 (98.2%)				
Thyroid disease	+	29	1 (3.4%)	28 (96.6%)	0.404	2.036	0.048	13.262
	-	1625	28 (1.7%)	1597 (98.3%)				
Cardiac disease	+	21	0 (0.0%)	21 (100.0%)	1.000	0.000	0.000	11.254
	-	1633	29 (1.8%)	1604 (98.2%)				
Pulmonary disease	+	20	0 (0.0%)	20 (100.0%)	1.000	0.000	0.000	11.873
	-	1634	29 (1.8%)	1605 (98.2%)				
Diabetes	+	109	1 (0.9%)	108 (99.1%)	1.000	0.502	0.012	3.097
	-	1545	28 (1.8%)	1517 (98.2%)				
Asthma	+	55	2 (3.6%)	53 (96.4%)	0.251	2.196	0.247	9.130
	-	1599	27 (1.7%)	1572 (98.3%)				
Smoking	+	76	1 (1.3%)	75 (98.7%)	1.000	0.738	0.018	4.594
	-	1578	28 (1.8%)	1550 (98.2%)				
Revision surgery	+	155	2 (1.3%)	153 (98.7%)	1.000	0.713	0.081	2.883
	-	1499	27 (1.8%)	1472 (98.2%)				

*BMI* Body mass index, *GFR* Glomerular filtration rate. * *P*<0.05Values are shown as the number of patients (%). Fisher's exact test

### Risk factors for *S. aureus*

In total, 445 cases (26.9 %) were positive for *S. aureus*. Therefore, the number of patients in the SA + group was 445, with 1209 in the SA − group. From univariate analysis, the proportion of female patients in the SA + group relative to all female patients (411 vs. 1464) was significantly higher than relative to all male patients (34 vs. 190) (28.1 % vs. 17.9 %, *p* = 0.003; OR: 1.790; 95 % CI: 1.205–2.724). No significant difference was seen with any other parameters (Table 2).

According to multivariate analysis, only female sex (*p* = 0.003; OR: 1.790; 95 % CI: 1.210–2.640) was an independent risk factor for the presence of *S. aureus* with a sensitivity of 0.924, specificity of 0.129.

**Table 2:** Univariate analysis of SA+ and SA- groups

							95% Confidence interval	
Parameter		n	SA+ (*n*=445)	SA- (*n*=1209)	*P*-value	Odds ratio	Lower	Upper
Sex	Female	1464	411 (28.1%)	1053 (71.9%)	0.003 *	1.790	1.205	2.724
	Male	190	34 (17.9%)	156 (82.1%)				
Age (years)	≥80	70	18 (25.7%)	52 (74.3%)	0.891	0.938	0.510	1.652
	<80	1584	427 (27.0%)	1157 (73.0%)				
BMI (kg/m^2^)	≥30	93	29 (31.2%)	64 (68.8%)	0.337	1.247	0.764	1.994
	<30	1561	416 (26.6%)	1145 (73.4%)				
Antibiotic use within 3 years	+	337	84 (24.9%)	253 (75.1%)	0.372	0.879	0.659	1.165
	-	1317	361 (27.4%)	956 (72.6%)				
Corticosteroid use	+	36	10 (27.8%)	26 (72.2%)	0.852	1.046	0.446	2.264
	-	1618	435 (26.9%)	1183 (73.1%)				
Albumin (g/dL)	<3.5	18	6 (33.3%)	12 (66.7%)	0.593	1.363	0.417	3.953
	≥3.5	1636	439 (26.8%)	1197 (73.2 %)				
GFR (mL/min)	<50	108	30 (27.8%)	78 (72.2%)	0.823	1.048	0.654	1.643
	≥50	1546	415 (26.8%)	1131 (73.2%)				
Brain disease	+	54	9 (16.7%)	45 (83.3%)	0.088	0.534	0.228	1.118
	-	1600	436 (27.3%)	1164 (72.7%)				
Thyroid disease	+	29	11 (37.9%)	18 (62.1%)	0.204	1.676	0.709	3.783
	-	1625	434 (26.7%)	1191 (73.3%)				
Cardiac disease	+	21	9 (42.9%)	12 (57.1%)	0.133	2.058	0.760	5.364
	-	1633	436 (26.7%)	1197 (73.3%)				
Pulmonary disease	+	20	5 (25.0%)	15 (75.0%)	1.000	0.905	0.256	2.638
	-	1634	440 (26.9%)	1194 (73.1%)				
Diabetes	+	109	32 (29.4%)	77 (70.6%)	0.576	1.139	0.718	1.771
	-	1545	413 (26.7%)	1132 (73.3%)				
Asthma	+	55	17 (30.9%)	38 (69.1%)	0.536	1.224	0.641	2.249
	-	1599	428 (26.8%)	1171 (73.2%)				
Smoking	+	76	16 (21.1%)	60 (78.9%)	0.289	0.714	0.380	1.273
	-	1578	429 (27.2%)	1149 (72.8%)				
Revision surgery	+	155	32 (20.6%)	123 (79.4%)	0.071	0.684	0.441	1.036
	-	1499	413 (27.6%)	1086 (72.4%)				

*BMI* Body mass index, *GFR* Glomerular filtration rate. *P<0.05Values are shown as the number of patients (%). Fisher's exact test.

### Calculation of costs

**Fig. 1:**
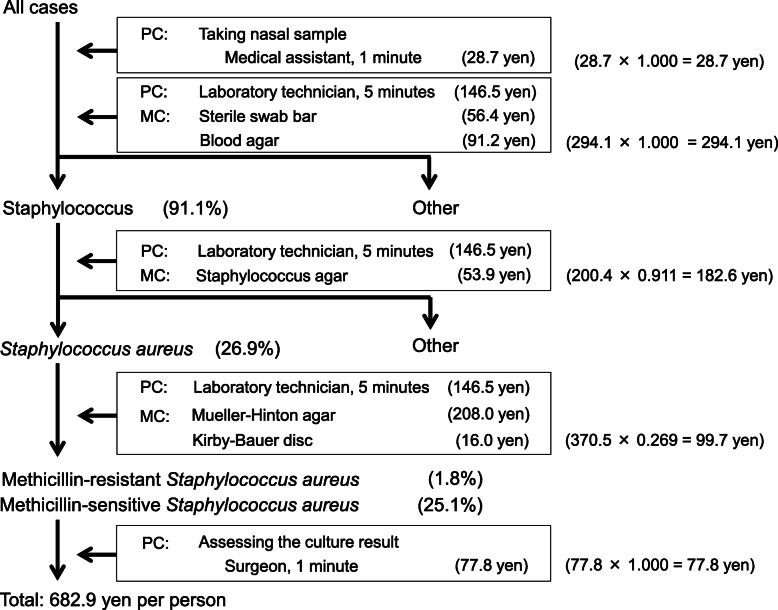
Calculation of screening cost. The average total cost for screening was 682.9 yen/person. PC: personnel cost. MC: material cost

The cost of each screening procedure was as follows: 28.7 yen to take nasal samples from all cases, 294.1 yen to isolate Staphylococcus strains from all samples, 200.4 yen to isolate *S. aureus* from Staphylococcus strains (91.1 % of all samples), 370.5 yen to divide *S. aureus* into MRSA and MSSA (26.9 % of all samples), and 77.8 yen to assess the culture results for all samples. Therefore, the total cost of screening, including personnel and material costs, was 28.7 + 294.1 + 200.4 × 0.911 + 370.5 × 0.269 + 77.8 = 682.9 yen per person (Fig. 1). The cost of mupirocin ointment in Japan is 1641.3 yen for each 3-g product. The cost for a medical assistant to spend 10 minutes instructing a patient on how to apply mupirocin ointment was 287.0 yen. Therefore, the total cost of decolonization was 1641.3 + 287.0 = 1928.3 yen per person.

When using the UD strategy, all patients need decolonization but none need screening. Therefore, the cost of the UD strategy is 1928.3 yen per person.

**Fig. 2:**
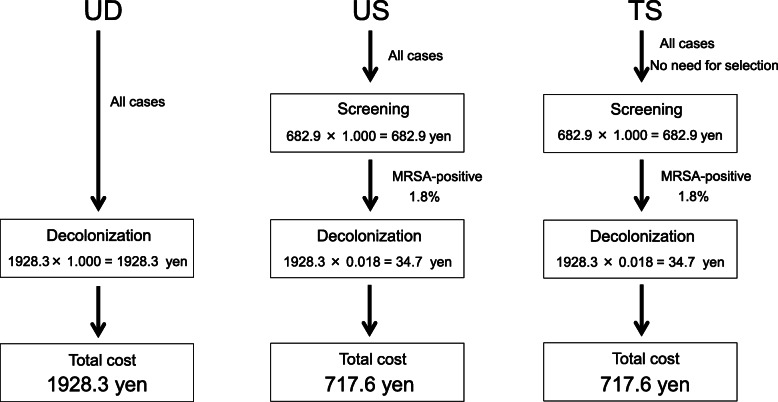
Average cost of each strategy for eradicating MRSA. The respective costs of UD, US, and TS were 1928.3, 717.6, and 717.6 yen/person. MRSA: methicillin-resistant *Staphylococcus aureus. *UD: universal decolonization. US: universal screening and target decolonization. TS: target screening and decolonization

For eradication of MRSA, because no risk factors for colonization were identified, it is impossible to select patients to undergo screening using the TS strategy. Mupirocin ointment was used as part of the US and TS strategies in patients who were MRSA-positive (29/1654 cases: 1.8 %). Therefore, the average cost of both US and TS is 682.9 + 1928.3 × 0.018 = 717.6 yen per person (Fig. 2).

**Fig. 3:**
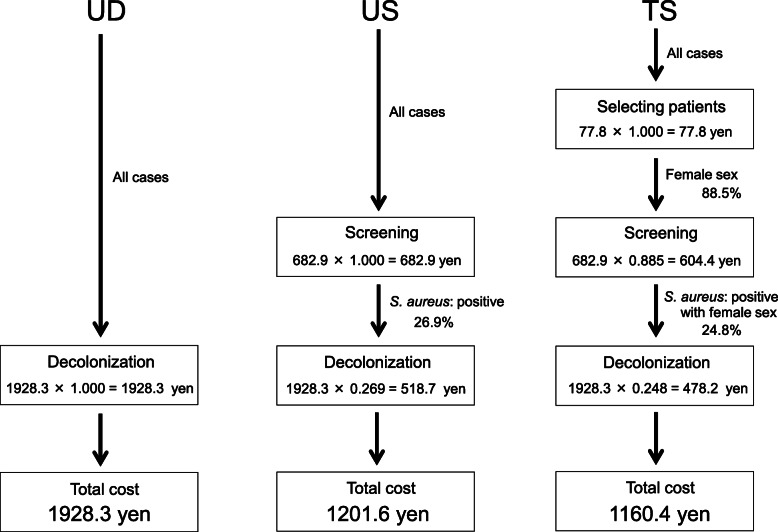
Average cost of each strategy for eradicating *S. aureus*. The respective costs of UD, US, and TS were 1928.3, 1201.6, and 1160.4 yen/person. *S. aureus*: both methicillin-resistant and methicillin-sensitive *Staphylococcus aureus*. UD: universal decolonization. US: universal screening and target decolonization. TS: target screening and decolonization

To eradicate *S. aureus* by the US strategy, all patients needed to be screened and treated by decolonization if *S. aureus*-positive (445/1654 cases: 26.9 %). Therefore, the average cost of the US strategy was 682.9 + 1928.3 × 0.269 = 1201.6 yen per person. As for the TS strategy, screened cases were selected according to risk factors by the surgeon (77.8 yen). All female patients were screened (1464/1654 cases: 88.5 %) and treated by decolonization if *S. aureus*-positive (411/1654 cases: 24.8 %). Therefore, the average cost of the TS strategy is 77.8 + 682.9 × 0.885 + 1928.3 × 0.248 = 1160.4 yen per person (Fig. 3).

### Incidence of SSI or PJI

A total of 1522 cases (92.0 %) were observed for more than 2 years of follow-up. There were 6 cases of superficial SSI, all of which were primary THA cases. Pathogens were 1 case of MRSA, 1 of methicillin-resistant coagulase-negative Staphylococcus, 2 of MSSA, and 2 of methicillin-sensitive coagulase-negative Staphylococcus. Among these patients, SSI pathogens of 1 MSSA case and 1 methicillin-sensitive coagulase-negative Staphylococcus case were matched with nasal bacteria. None of these six SSI cases were MR + and only 1 case was SA+. There were no cases of deep incisional SSI. Other than these, there were no cases of PJI during the follow-up period.

## Discussion

In this study, no risk factors for MRSA were identified and the risk factor for *S. aureus* carriage was female sex only. As for *S. aureus* carrier status, specificity of female sex as a risk factor was 0.129. Because of low specificity, implementing the TS strategy considering this risk factor would require screening a considerable number of patients—amounting to 88.5 % of all patients—with 4 of 29 (13.8 %) MRSA-positive cases and 34 of 445 (7.6 %) *S. aureus*-positive cases being overlooked. Thus, this parameter is not useful and no suitable risk factor for the TS strategy was identified.

Previous studies have attempted to clarify various risk factors, including male sex, white race, obesity, asthma [12], diabetes [13], and renal disease [14] for both MSSA and MRSA colonization, but opinions remain divided. While smoking was described as a risk factor in one study [15], it was found to have no relationship with *S. aureus* colonization in other studies [1, 12]. De Wouters et al. reported that although Belgian guidelines recommend TS for MRSA carriage on admission for high-risk populations only (age > 80 years; inpatient admission in the previous 6 months; past history of MRSA colonization; living in a nursing or residential home; exposure to invasive devices; chronic wounds or skin lesions; working in health care; and being in contact with farm animals), there was no correlation between identified MRSA carriers and these risk factors [16]. No risk factors for MRSA were identified in this study. Moreover, when female sex was applied as a risk factor for TS, the average cost of US vs. TS per person to decolonize *S. aureus*-positive cases was 1201.6 vs. 1160.4 yen; thus, TS could reduce costs by 41.2 yen (3.6 %) compared with US. As such, no useful predictive factors that enable the successful implementation of TS were identified and the strategy was also not that cost-effective.

The UD strategy is advocated in clinical units with a high risk of MRSA infection, such as intensive care units and emergency units, because it can protect patients during a period of vulnerability to infection and it can prevent delayed decolonization pending the results of screening [7]. However, in cases of elective surgery like arthroplasty, there is no urgency that requires UD, because patients are not particularly vulnerable and the waiting period for screening results is irrelevant. Moreover, UD as empiric therapy is not recommended because of the risk of increasing bacterial resistance [11]. Prior mupirocin use was reported to increase the risk of mupirocin resistance in MRSA carriers by 9 fold [17]. According to Graber and Schwartz, failure of decolonization may be the result of increased mupirocin resistance [18]. Another disadvantage of UD is financial burden. In this study, mupirocin ointment at 1928.3 yen/product is needed to implement UD for all patients when including personnel costs. In the United States, Stirton et al. reported the cost of empiric treatment with mupirocin for all patients as an estimated $24.65 per patient (equivalent to 2711.5 yen at 110 yen to 1 US Dollar), which included the personnel costs for instruction on mupirocin application [19].

The incidence of deep SSI in THA was reported as 1.1 % [20], and the prevalence of revision THA due to PJI was reported as 0.4 % following primary procedures and 1.6 % following revision hip arthroplasty [21]. Bozic and Ries reported a longer duration of hospitalization with revision arthroplasty for infection than with aseptic loosening (28.2 vs. 8.1 days, *p* < 0.001) as well as higher total hospital cost ($96,166 vs. $34,866, *p* < 0.001) and higher outpatient charges ($48,348 vs. $16,411, *p* < 0.001) [22]. The cost of care for treatment of deep SSI caused by methicillin-resistant strains was estimated at $107,264 compared with $68,053 when caused by sensitive strains [2]. Therefore, revision arthroplasty due to infection is very costly.

Regarding SSI after total joint arthroplasty, MRSA and MSSA were reported as the most common pathogens [23]. Colonization of the nares occurs at higher rates compared with other body surfaces, and 65 % of cases of MRSA colonization were detected in the nares [24]. There are three general sources of infection: endogenous, exogenous, and hematogenous [25]. Nasal carriage of *S. aureus* is thought to be endogenous to patients and is a well-established risk factor for SSI or PJI. The risk of SSI following orthopedic surgery was reported as 6.9 times higher among patients with preoperative MRSA nasal colonization [26] and 2.8 times higher with preoperative MSSA nasal colonization [27]. Thus, because nasal carriage of *S. aureus* is an important factor for infection, the importance of intranasal decolonization cannot be overemphasized. Mupirocin can eliminate *S. aureus* nasal carriage in healthy persons for up to 3 months, with a corresponding effect on hand carriage [28]. Treating nasal carriage usually leads to eliminating *S. aureus*, including MRSA, from other areas of the body [29].

The effectiveness of US for reducing SSI following THA has been widely reported. According to Nixon et al., implementing the US strategy for MRSA eradication reduced the cost of care, considering the enormous costs incurred by revision arthroplasty and prolonged admission due to infection [9]. Hacek et al. implemented US for 1495 cases of total joint arthroplasty with decolonization for *S. aureus*-positive patients and reported reduced SSI compared with 583 non-screened or decolonized control cases (0.77 % vs. 1.7 %, *p* ≤ 0.1) [4]. Pofahl et al. reported that the SSI rate of US with decolonization for MRSA-positive patients was significantly lower than that for non-intervention controls (0 % vs. 0.30 %, *p* = 0.04) for total joint arthroplasty [5]. Thus, implementing US can reduce the SSI rate, costs related to revision arthroplasty, and hospitalization duration.

In the present study, there were 6 cases of superficial SSI but no cases of deep incisional SSI or PJI. Among the six superficial SSI cases, 1 was SA+, 5 were SA−, and none were MR+. Although the number of SSIs was overall very low, considering the SA + rate of 26.9 % and SA − rate of 73.1 %, the number of SSIs in the SA + group can be considered very low despite the different risk factors. We decolonize only MRSA-positive patients with mupirocin and treat both nares twice daily for 5 days. At present, we believe that this method is adequate because of the low SSI rate in the MR + and SA + groups in this study. However, several reports recommend decolonization in patients who are *S. aureus*-positive [4, 28] but whether this should be done remains controversial. Further research would be needed to identify patients in whom decolonization should be performed.

In this study, no risk factors were identified for MRSA carriers and only female sex was identified, albeit with low specificity, for *S. aureus* carriers. Thus, no risk factors that could help with TS were identified. Also, TS was determined not to be as cost-effective as US. UD, which is suitable for intensive care units and emergency rooms, is a more expensive strategy than US for eradicating both MRSA and *S. aureus*. US would be a more cost-effective strategy than UD for THA patients whose screening results can be waited for. Therefore, overall, US is considered to be the most cost-effective strategy with reduced sampling error rates for THA patients.

There are several limitations in this study. First, the study population included few patients with extremely high risk such as age > 90 years, poorly controlled diabetes, currently undergoing dialysis, and those with human immunodeficiency virus infection. Second, the study had a retrospective design, which means that there was no information available on whether there was a recent history of parenteral antibiotic therapy [30], homosexual activity [31], a recent stay in a nursing home or emergency ward, living with a health care worker, or being a health care worker [32], all of which are putative risk factors for nasal carriage. Third, we attempted to identify risk factors for nasal bacterial carriage in a Japanese population, but different results might be obtained elsewhere. Further investigation is needed to clarify the risk factors for other countries. Fourth, in the calculation of costs, personnel costs would differ considerably depending on the methods used and the insurance systems in place in each country. Fifth, we relied on a nasal culture swab method for determining the presence of MRSA and *S. aureus*; however, this method has low sensitivity and might introduce false-negative results [6]. For high-risk populations, it is desirable to use more reliable methods, such as polymerase chain reaction, despite their higher cost. Sixth, in order to determine which strategy is the most appropriate, ideally there should have been three intervention cohorts (UD, US, and TS) and a non-intervention cohort (without decolonization). However, there was no UD, TS, or non-intervention cohort in this retrospective study, so we could not provide any comparative data on the incidence of SSI or PJI in these groups. Seventh, although some researchers have emphasized the effectiveness of a bundle protocol consisting of self-administered nasal mupirocin and bathing with chlorhexidine gluconate [33, 34], others have used only nasal mupirocin ointment [4, 6]. In this study, we simulated nasal decolonization alone. Moreover, although the nose is considered to be the most important carriage site [28, 29], further investigation is needed to determine whether it is necessary to obtain screening swabs from other body sites. Finally, there were only 6 cases of superficial SSI, 4 of which the causative pathogens did not match the nasal bacteria, and there were no cases of PJI in the 2-year follow-up period. Therefore, from the results of this study, it is unclear whether bacteria in the nose affect SSI or PJI. Further investigation is needed to clarify the relationship between nasal bacterial carriage and infection in THA.

## Conclusions

No predictive factors for nasal carriage of MRSA and *S. aureus*, which are useful for implementing the TS strategy, were identified. TS reduced the cost of eradicating *S. aureus* by only 3.6 % compared with US. The cost of UD for eradicating both MRSA and *S. aureus* is greater than that of US or TS. Taking these findings together, the US strategy is considered to reduce sampling errors and to be the most cost-effective strategy to implement in THA patients.

## Data Availability

The datasets used and/or analyzed during the current study are available from the corresponding author on reasonable request.

## Abbreviations

THA: Total hip arthroplasty
PJI: Periprosthetic joint infection
MRSA: Methicillin-resistant *Staphylococcus aureus*
MSSA: Methicillin-sensitive*Staphylococcus aureus*
*S. aureus*: Both MRSA and MSSA
SSI: Surgical site infection
UD: Universal decolonization
US: Universal screening and target decolonization
TS: Target screening and decolonization
BMI: Body mass index
GFR: Glomerular filtration rate
OR: Odds ratio
CI: Confidence interval
